# 1151. Assessing Vaccine Hesitancy and Logistical Barriers to Undergraduate Students Receiving a Flu Vaccine

**DOI:** 10.1093/ofid/ofad500.992

**Published:** 2023-11-27

**Authors:** Casey Benzaken, Tina Q Tan, Ravi Jhaveri, Leena B Mithal

**Affiliations:** Feinberg School of Medicine, Chicago, Illinois; Feinberg School of Medicine, Northwestern University, Chicago, Illinois; Ann & Robert H. Lurie Children's Hospital of Chicago, Chicago, Illinois; Ann and Robert H. Lurie Children's Hospital of Chicago, Chicago, Illinois

## Abstract

**Background:**

Rates of annual flu vaccine receipt among college students remains significantly lower than the 2030 Healthy People target of > 70%. College students are at increased risk of contracting influenza due to close living and dining conditions, and illness due to influenza can have a negative impact on school attendance and academic achievement. This study assessed the extent to which vaccine hesitancy and logistical factors impacted college students’ ability to receive a flu vaccine.

**Methods:**

This study consisted of three surveys administered through REDCap. During the 2021-22 and 2022-23 flu seasons, we administered a baseline survey to assess intention to receive a flu vaccine and logistical barriers that students face in receiving a flu vaccine. We also assessed vaccine hesitancy using a modified Parental Attitudes about Childhood Vaccines (PACV) questionnaire. One month later, we administered a follow-up survey to assess whether students had received a flu vaccine and to rank the importance of several factors in their decision-making.

**Figure:**
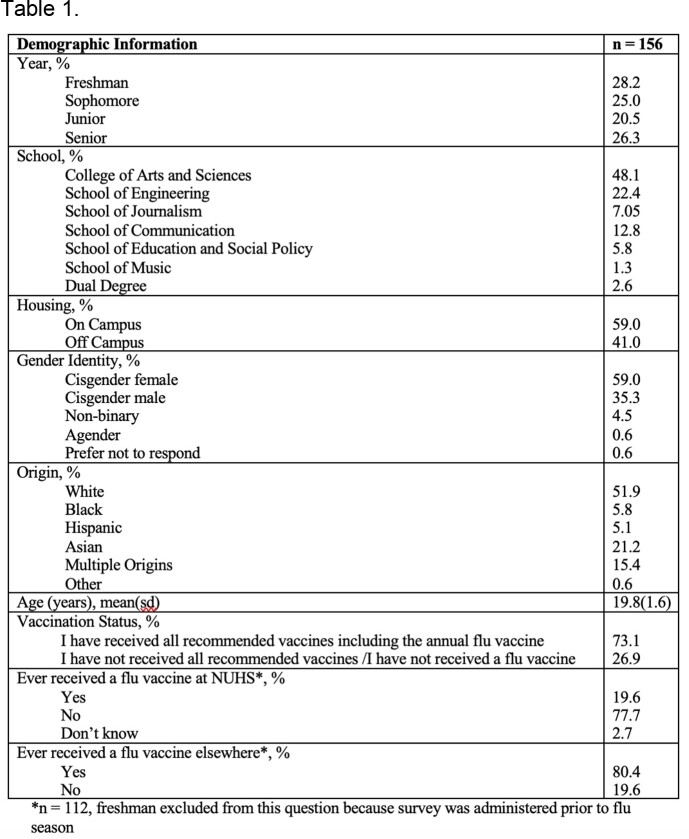

Sociodemographic information of respondents to the baseline survey.

**Results:**

We had a total of 156 participants in the baseline survey and 97 participants in the follow-up survey. Sociodemographic information can be seen in Table 1. There was a self-reported flu vaccination rate of 63.9%. Only 4.5% of participants scored ≥50 for their adapted PACV survey, indicating that a small proportion of students are flu vaccine hesitant. Individuals who self-reported that they typically receive a flu vaccine yearly had statistically significantly (p < 0.0001) lower adapted PACV scores (11.0[9.5]) compared to individuals who self-reported that they do not typically receive a flu vaccine annually or have only received the vaccines needed to enter school (31.3[21.3]). Among individuals who did not receive a flu vaccine, the top three concerns were easiness of forgetting (78.6%), inconvenience (57.2%), and lack of time (49.9%).

**Conclusion:**

Understanding undergraduate barriers to receiving flu vaccines is critical in designing interventions that increase flu vaccine uptake in this population. Our study found that logistical barriers, rather than vaccine hesitancy, was the limiting factor in preventing college students from receiving flu vaccines.

**Disclosures:**

**Tina Q. Tan, MD**, GSK: Advisor/Consultant|GSK: Grant/Research Support|Iliad: Advisor/Consultant|Merck: Advisor/Consultant|Moderna: Advisor/Consultant|Novavax: Advisor/Consultant|Pfizer: Advisor/Consultant|Sanofi Pasteur: Advisor/Consultant|Sanofi Pasteur: Grant/Research Support **Ravi Jhaveri, MD**, AbbVie: Grant/Research Support|AliOS: Grant/Research Support|AstraZeneca: Advisor/Consultant|Gilead: Grant/Research Support|MedImmune: Advisor/Consultant|Merck: Grant/Research Support|Saol Therapeutics: Advisor/Consultant|Seqirus: Advisor/Consultant

